# Is carbohydrate needed to further stimulate muscle protein synthesis/hypertrophy following resistance exercise?

**DOI:** 10.1186/1550-2783-10-42

**Published:** 2013-09-25

**Authors:** Vandré Casagrande Figueiredo, David Cameron-Smith

**Affiliations:** 1 The Liggins Institute, Faculty of Medical and Science Health, University of Auckland, 85 Park Road, Grafton, Private Bag 92019, Auckland 1023, New Zealand

**Keywords:** Leucine, Insulin, Glycemic index, Skeletal muscle, Dietary supplements, Whey protein, Lean body mass

## Abstract

It is now well established that protein supplementation after resistance exercise promotes increased muscle protein synthesis, which ultimately results in greater net muscle accretion, relative to exercise alone or exercise with supplementary carbohydrate ingestion. However, it is not known whether combining carbohydrate with protein produces a greater anabolic response than protein alone. Recent recommendations have been made that the composition of the ideal supplement post-exercise would be a combination of a protein source with a high glycemic index carbohydrate. This is based on the hypothesis that insulin promotes protein synthesis, thus maximising insulin secretion will maximally potentiate this action. However, it is still controversial as to whether raising insulin level, within the physiological range, has any effect to further stimulate muscle protein synthesis. The present commentary will review the evidence underpinning the recommendation to consume carbohydrates in addition to a protein supplementation after resistance exercise for the specific purpose of increasing muscle mass. The paucity of data will be discussed, thus our conclusions are that further studies are necessary prior to any conclusions that enable evidence-based recommendations to be made.

## Background

In the past decade significant progress has been made in unravelling the mechanisms that regulate the complex pathways that couple gene expression to protein synthesis. Emerging from these studies has been the influence of amino acids, most predominately leucine, on protein synthesis. Leucine, over and above being a necessary amino acid in protein synthesis, also potentiates the activity of the key kinases regulating translation initiation. Far from being the only determinate of protein synthesis, leucine along with energy status, mechano-sensing, ionic and hormonal mediators all converge to dictate the rate of protein synthesis. Insulin also plays an important role in protein synthesis, as a potent stimulator of PI-3K/Akt/mTOR axis, coupling growth with nutritional availability.

In a recent review by Stark et al. [1] published in the *Journal of the International Society of Sports Nutrition*, it was stated that fast-acting carbohydrates (e.g., glucose or maltodextrin) should be consumed together with proteins after resistance exercise to promote muscle hypertrophy. The two primary reasons cited for the necessity of adding carbohydrates to the post exercise meal/supplement were; 1), acutely, there is a synergistic effect of insulin and leucine on protein synthesis; and, 2) chronically, the addition of carbohydrate to a protein supplement would increase Lean Body Mass (LBM) to a greater extent than when protein is consumed alone. These assumptions require careful analysis, given the almost total absence of clinical data and the unsupported statements that have been made.

### Does leucine require insulin to stimulate protein synthesis?

It is physiologically relevant to state that “leucine cannot modulate protein synthesis as effectively without the presence of insulin” as Stark et al. [1] claimed. However, the cited supportive data [2,3] are both cell culture in vitro models where it is possible to exclude insulin entirely from the treatment conditions. Results from cell culture studies are therefore not necessarily transferable to the *in vivo* conditions, without consideration of the differences. In one of these studies the cells were deprived of serum overnight (12+ hours) prior to stimulation with insulin [2]. Both studies [2,3] compared insulin treated cells with untreated cells. This contrasts the physiological state, in which even short-term (overnight) fasting conditions have low, but measurable levels of circulating insulin (~5 mU/L). At these low levels, protein synthesis can be elicited by amino acids [4]. More importantly, increasing insulin levels more than 30 times over fasting levels has no further effect on protein synthesis even while aminoacidemia is kept at high levels [4]. Thus, technically it is true that insulin is needed to increase protein synthesis when amino acids delivery are increased, however even very low levels of insulin are able to act in concert with leucine to enable protein synthesis. Moreover, it should be noted that leucine ingestion has the ability to stimulate insulin secretion [5,6] and that the majority of protein supplementation studies report a marked increase in circulating insulin concentrations, at a minimum 2–3 fold above fasting values [7,8].

### Does insulin act to inhibit protein degradation?

Given that at physiological concentrations, increased insulin is unlikely to augment protein synthesis *in vivo*, it is also necessary to consider whether this also applied for protein degradation. Indeed, Børsheim et al. [9] demonstrated that carbohydrate supplementation (100 g) alone following resistance exercise is capable of improving net muscle protein balance through reduction at protein degradation rates rather than through increasing protein synthesis. However, the resultant small increase in insulinemia due to protein ingestion alone is also sufficient to inhibit the increased protein breakdown measured after resistance exercise [10].

### Absence of clinical data on muscle gain

It has been further stated by Stark et al. [1] that “(…) studies using protein sources with a carbohydrate source tended to increase LBM more than did a protein source alone” (herein cited as references 11, 12, 13, 14, 15, 16). This fails to adequately reflect the data within these cited studies. The majority of those studies compared the co-ingestion of protein and carbohydrate versus carbohydrate alone [11,12] or versus a different source of protein whilst maintaining similar amounts of carbohydrates [13-15]. Moreover, the last cited study [16] analysed the impact of supplementation timing, not supplement composition. To date there are no clinical studies comparing the impact of the co-ingestion of carbohydrate-protein with just protein supplement on LBM. Interestingly, Wilkinson et al. [14] and Hartman et al. [13] both compared different sources of protein (milk versus soy) which also contained appreciable levels of carbohydrate. Both beverages had similar amounts of carbohydrate but the glycemic index (GI) differed: soy group contained maltrodextrin while milk group had lactose (as expected). Yet it was the lower GI supplement (milk) which generated the greatest net gain in lean mass [13] and higher fractional synthesis rate [14]. In these studies, at least, GI was not positively associated with muscle gains.

To date only three studies [10,17,18] have addressed the impact of combined carbohydrate with protein/amino acids versus protein/amino acids alone on acute protein synthesis in young adults. These studies demonstrate that adding carbohydrate to a protein dose that alone is known to maximally stimulate protein synthesis (20-25 g of high-quality protein rich in leucine) has no additive or synergic effect on muscle protein synthesis and breakdown. The same result has recently also been demonstrated in older subjects [19]. Converging with those data, the addition of 30 g or 90 g of carbohydrates to 20 g of essential amino acids produces the same effect on protein synthesis and protein breakdown, regardless the great difference in insulinemia in both groups [20]. Insulin seems to only further increase protein synthesis at pharmacological doses [21], which means that it is not achievable by carbohydrate supplementation.

There remain valid reasons for the inclusion of carbohydrates into protein supplements that are to be consumed following resistance exercise. These included the maximization of glycogen restoration, especially when the time period between exercise sessions is short [22]. However, based on the available clinical data, there is no evidence that the addition of carbohydrates to a protein supplement will increase, acutely, muscle protein synthesis and, chronically, LBM to a greater extent than protein alone, which is in contrast to the statements of Stark and colleagues [1].

## Conclusion and perspectives

There is a growing body of literature analysing the impact that co-ingestion of protein-carbohydrate versus carbohydrate alone has on protein synthesis. However, there are far fewer studies examining the actions of co-ingested carbohydrate-protein compared to protein alone. More importantly, no chronic study has addressed the effects of adding carbohydrate to protein compared to protein alone on muscle hypertrophy.

In conclusion, whilst it cannot be excluded that carbohydrate addition may provide benefits for recovering athletes, on the basis of available data, no further beneficial actions of carbohydrates, irrespective of GI, are evident concerning muscle hypertrophy when a protein supplement that maximally stimulate muscle protein synthesis is ingested. Further studies are required before conclusions and recommendations can be made.

## Abbreviations

LBM: Lean body mass; GI: Glycemic index; mU/L: milliunits per liter; PI-3K: Phosphoinositide 3-kinase; Akt: Protein kinase B; mTOR: Mammalian target of rapamycin.

## Competing interests

The authors declare that they have no competing interests.

## Authors’ contributions

VCF and DCS wrote the manuscript. Both authors read and approved the final version.
